# Supplementary material for: Ultrathin crown ether-based polyamide membrane for ion-ion separations
Source: Nat Commun. 2026 Mar 20;17:4263. doi: 10.1038/s41467-026-70431-1 (PMC13168650; doi:10.1038/s41467-026-70431-1)
Supplement: Supplementary file 1 — Supplementary Information [file 41467_2026_70431_MOESM1_ESM.pdf]

Supplementary Information for "Ultrathin crown ether-based polyamide membrane for ion-ion separations"

Luis Francisco Villalobos[1*], Junwei Zhang[2], Junwoo Lee[2,3], Alex T. Hall[4], Ryan M. DuChanois[5], Camille Violet[2], John Cumings[4], Mingjiang Zhong[2,6], Menachem Elimelech[5,7]

[1] Mork Family Department of Chemical Engineering and Materials Science, University of Southern California, Los Angeles, CA, 90089, United States
[2] Department of Chemical and Environmental Engineering, Yale University, New Haven, CT, 06520, United States
[3] Department of Molecular Science and Technology, Ajou University, Yeongtong-gu, Suwon-si, Gyeonggi-do 16499, Republic of Korea
[4] Department of Material Science and Engineering, University of Maryland, College Park, MD, 20742, United States
[5] Department of Chemical and Biomolecular Engineering, Rice University, Houston, TX 77005, United States
[6] Department of Chemistry, Yale University, New Haven, CT, 06520, United States
[7] Department of Civil and Environmental Engineering, Rice University, Houston, TX 77005, United States

[*] Email: lf.villalobos@usc.edu

Supporting Information

Supplementary Note 1: Solubility of DAB18C6 and reaction conditions. DAB18C6 exhibited solubility in water only at low pH, attributed to the protonation of its amine groups, which increased its hydrophilicity. Protonated amines, however, do not effectively react with acyl chlorides due to the positive charge on the nitrogen, which eliminates the lone pair of electrons necessary for nucleophilic attack. Nevertheless, a small amount of neutral amine, in equilibrium with its protonated form, can still react with acyl chlorides. As the neutral amine is consumed, the equilibrium shifts to regenerate more of it, allowing the reaction to proceed gradually. Leveraging this principle, we attempted to form a polyamide film at the interface of a 0.4% (w/v) DAB18C6 solution in 0.03 M HCl and a 0.2% (w/v) TMC solution in hexane. The reaction was slow; a visible film did not appear until approximately 20 minutes after the solutions were contacted. After 1 hour of reaction, the film formed at the interface was transferred to a water bath. However, the film lacked mechanical stability and shattered upon floating in the water. Imaging one of the larger fragments supported on AAO substrate revealed additional defects, likely caused by the transfer and drying processes (Supplementary Fig. 2A,B).

In contrast, using a co-solvent system where DAB18C6 was more readily soluble resulted in the formation of more robust films. Specifically, a target concentration of 0.2% (w/v) DAB18C6 in a solvent mixture of 20% DMF and 80% water (v/v) was prepared. Contacting this solution with a 0.2% (w/v) TMC solution in hexane led to the immediate precipitation of a thin film at the interface. These films demonstrated significantly improved mechanical stability and could be successfully transferred to a variety of porous substrates, such as AAO (Supplementary Fig. 2C) and PVDF (Supplementary Fig. 2D).

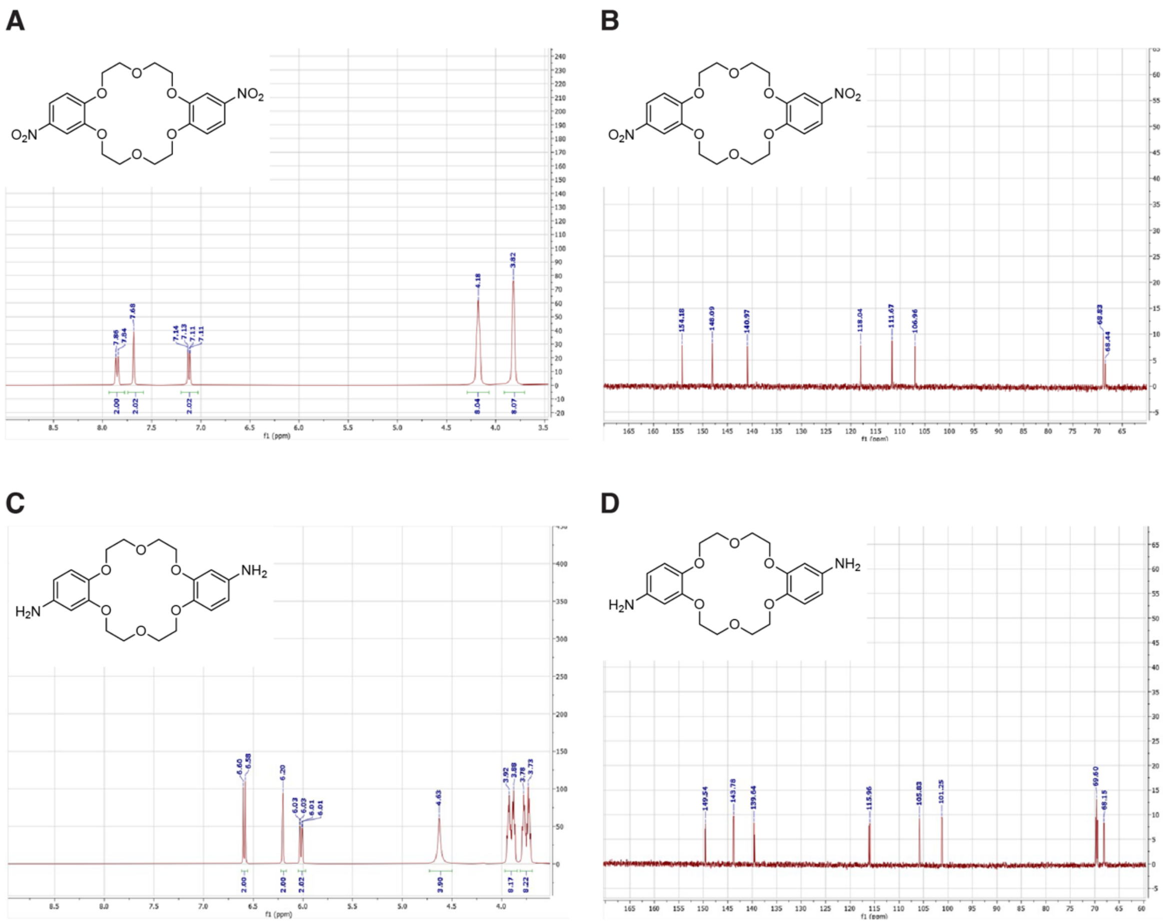

Supplementary Figure 1. A) ^{1}H NMR spectrum of trans-di(nitrobenzo)[18]crown-6; B) ^{13}C NMR spectrum of trans-di(nitrobenzo)[18]crown-6; C) ^{1}H NMR spectrum of trans-di(aminobenzo)[18]crown-6; D) ^{13}C NMR spectrum of trans-di(aminobenzo)[18]crown-6. Spectra were recorded in chloroform-d at 400 MHz (^{1}H) and 100 MHz (^{13}C).

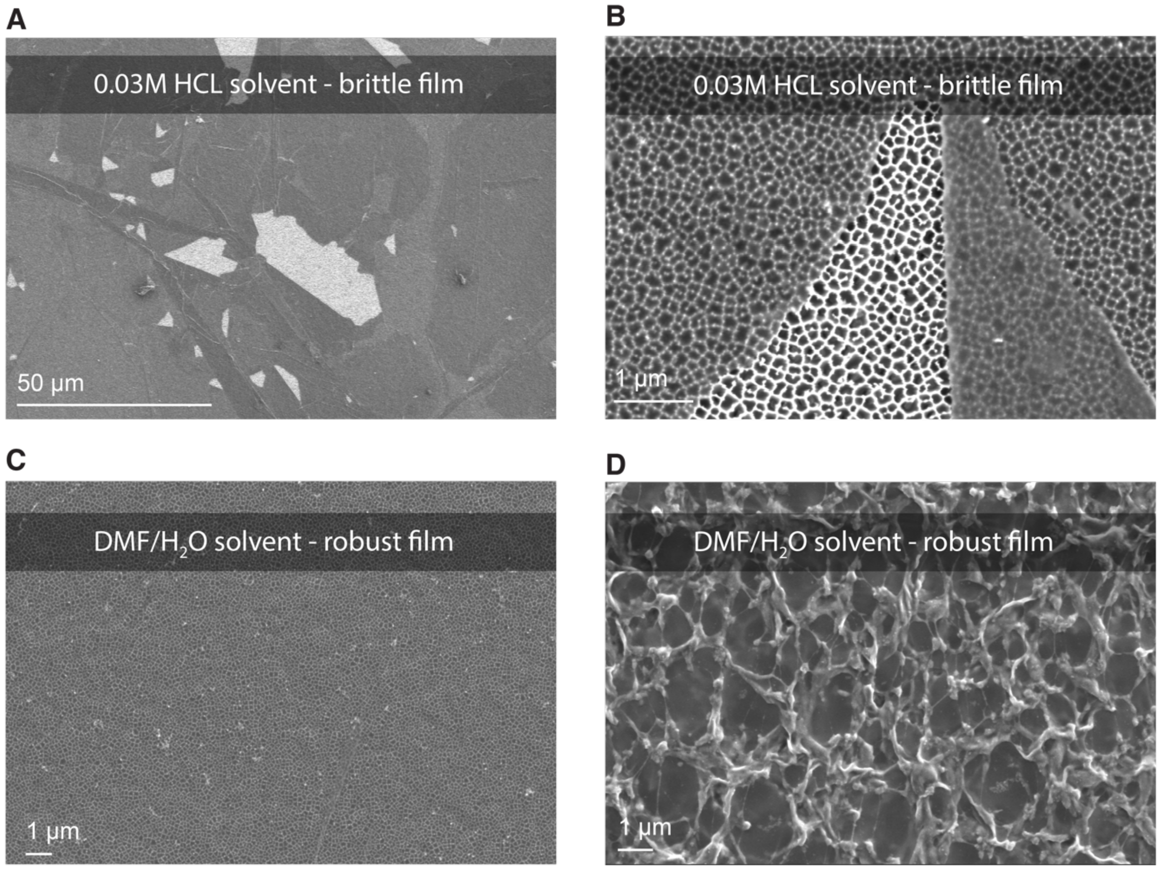

Supplementary Figure 2. A, B) SEM images of the crown ether film formed under acidic conditions to dissolve the DAB18C6 monomer (0.4 wt.% DAB18C6 in 0.03 M HCl and 0.2 wt.% TMC in hexane, with a reaction time of 60 minutes). The film shattered upon floating on water, and visible defects were observed after transferring a piece of it to an AAO porous support. C, D) SEM images of the crown ether film formed using DMF as a co-solvent to dissolve the DAB18C6 monomer (0.2 wt.% TMC in hexane and 0.2 wt.% DAB18C6 in a 20/80 v/v DMF-water mixture, with a reaction time of 5 minutes). These films retained their shape upon floating on water, and no defects were observed after transferring them to either an AAO porous support (Supplementary Fig. 2C) or a PVDF porous support with larger pores (Supplementary Fig. 2D).

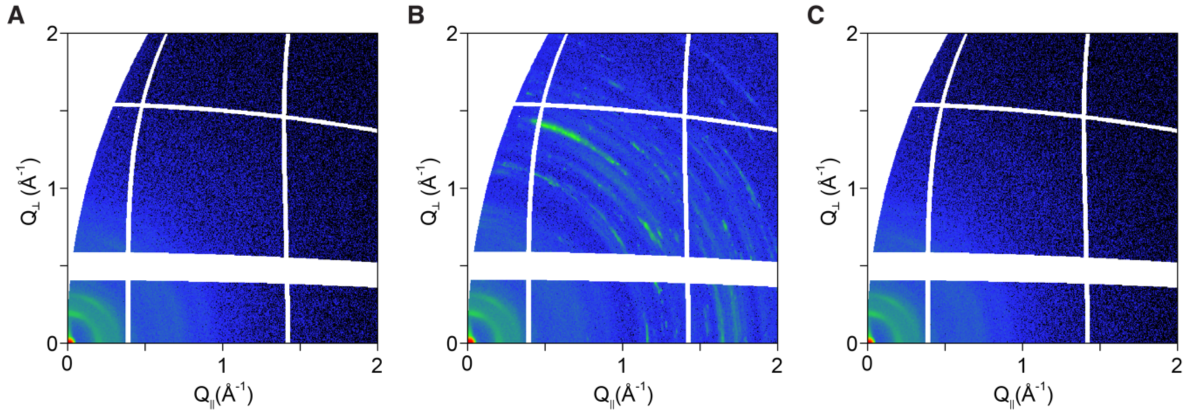

Supplementary Figure 3. GI-WAXS two-dimensional images of: (A) a bare Si wafer, (B) the DAB18C6 monomer deposited by drying a drop of a DMF solution on the surface of the Si wafer, and (C) the prepared crown-ether polyamide film on a Si wafer.

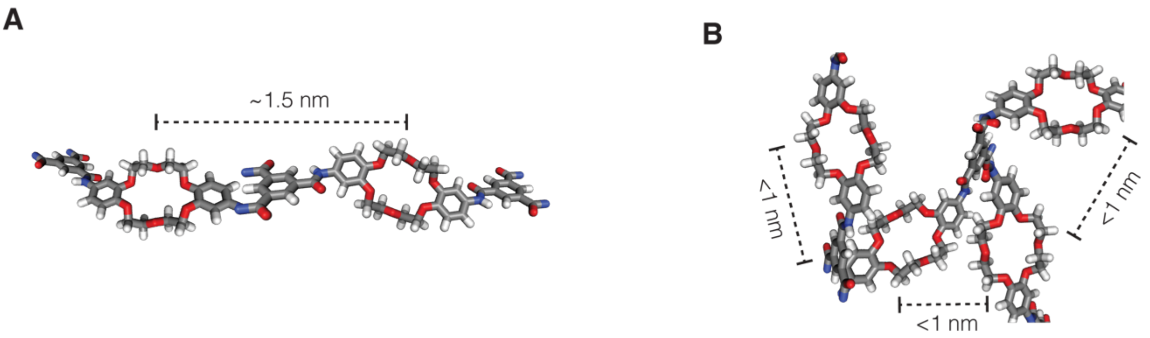

Supplementary Figure 4. Schematic illustrating the approximate distance between two crown ether centers in the extreme case of a fully stretched polymer chain (A). In contrast, within the randomly arranged structure of the prepared films, the average distance between crown ether sites is expected to be much shorter (B).

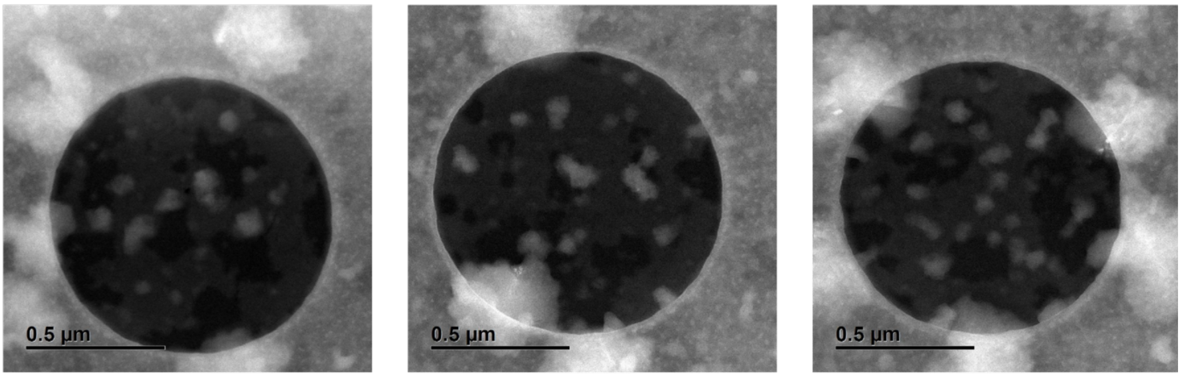

Supplementary Figure 5. Cryogenic LAADF-STEM images revealing the presence of crystallites in the polyamide matrix.

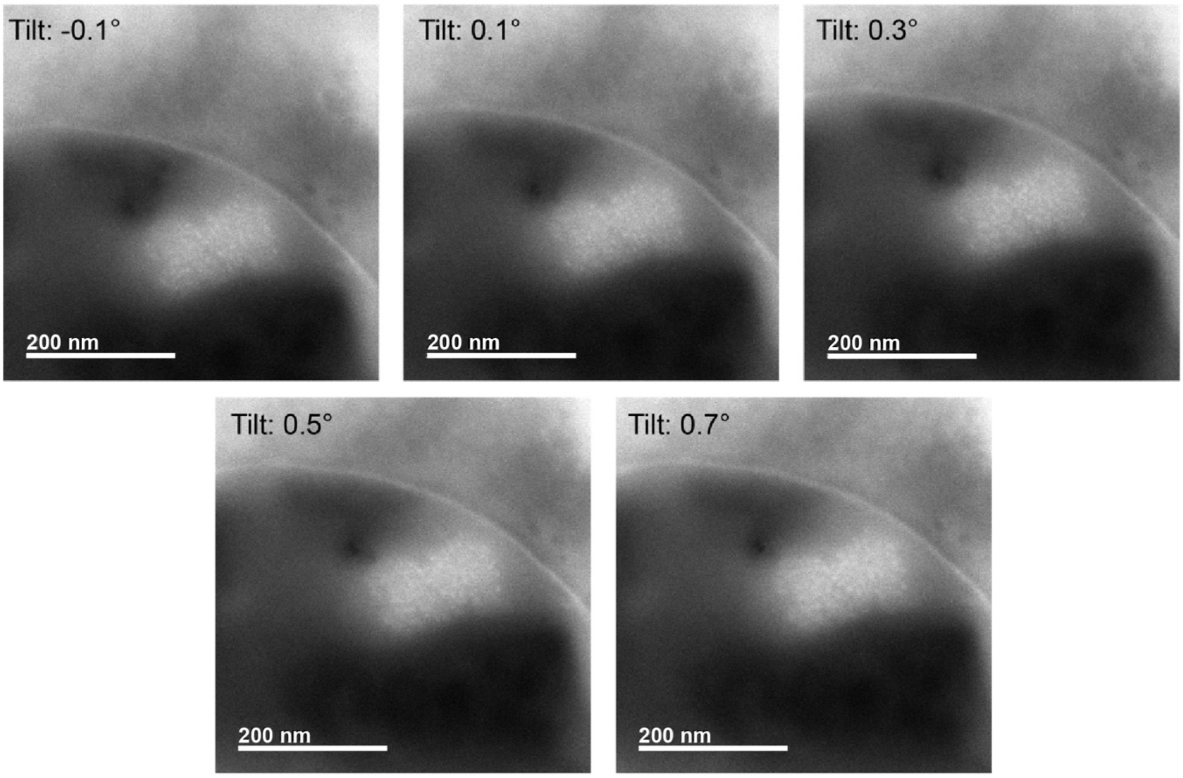

Supplementary Figure 6. Cryogenic LAADF-STEM images of the membrane at different tilt angles. Tilting the sample reveals contrast fluctuations characteristic of polycrystalline regions, providing strong evidence of crystalline regions within the membrane.

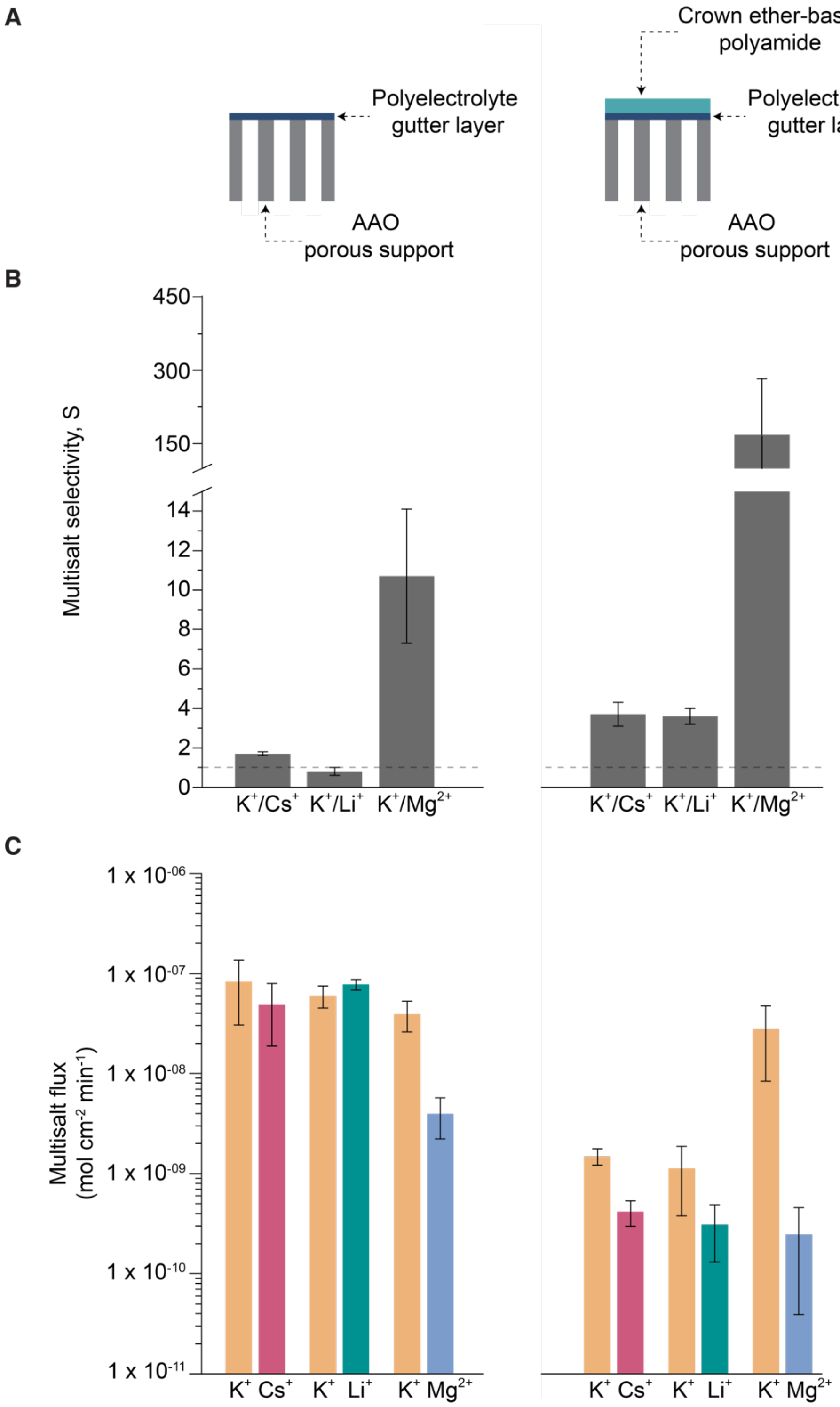

Supplementary Figure 7. A) Schematic of the support used in our measurements, shown before (left) and after (right) depositing the polyamide crown ether-based selective layer. AAO refers to anodic aluminum oxide, and the polyelectrolyte gutter layer was fabricated via layer-by-layer deposition of polyacrylic acid/polyethylenimine/polyacrylic acid. The membranes were mounted between the feed and receiving chambers of a diffusion cell, temperature-controlled using a water jacket. All experiments were conducted at 25 °C with binary cation mixtures (chloride salts) in the feed and deionized water as the receiving solution. B) Average selectivity of K^+ over other cations (Cs^+, Li^+, or Mg^{2+}) for the bare support (left) and the supported crown ether-based membrane (right) under three different multi-salt feed conditions: 0.1 M KCl combined with 0.1 M CsCl, LiCl, or $MgCl_2$ (three membranes each). C) Average cation flux for the same experiments as in B, comparing the bare support (left) and the supported crown ether-based membrane (right). Data are presented as mean ± SD from n = 3 independent measurements (panels B and C).

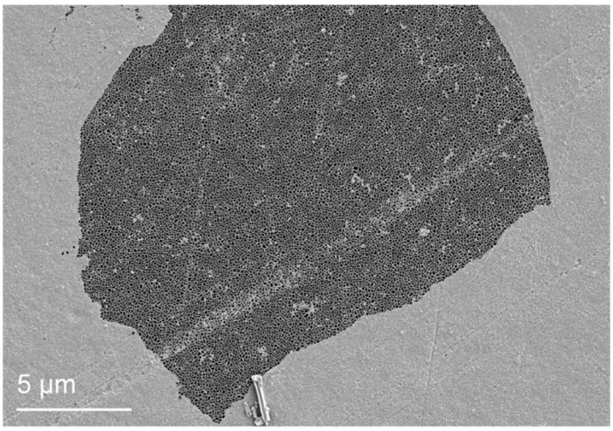

Supplementary Figure 8. SEM image of a composite crown ether-based polyamide membrane showing defects introduced during transfer and testing. These defects resulted in the absence of monovalent ion selectivity and a K^+ flux comparable to that of the bare support.

Supplementary Table 1. Mixed ion transport experiment data for the K^+/Cs^+ system. Only data corresponding to membranes exhibiting a K^+ flux at least one order of magnitude lower than that of the bare polyelectrolyte-coated AAO support (values above the gray bar) are included in the analysis, as low K^+ flux is used as an indicator of a continuous, pristine crown-ether selective layer governing transport rather than defect-mediated leakage.

Measurement	K^+ Flux (mol cm^{-2} min^{-1})	Cs^+ Flux (mol cm^{-2} min^{-1})	K^+/Cs^+
1	1.15E-09	2.6E-10	4.4
2	1.42E-09	4.00E-10	3.6
3	1.57E-09	5.40E-10	2.9
4	1.81E-09	4.70E-10	3.9
5	2.63E-08	8.39E-09	3.1
6	3.67E-08	1.41E-08	2.6
7	5.69E-08	2.12E-08	2.7
8	1.21E-07	5.60E-08	2.2
9	1.75E-07	9.12E-08	1.9
10	1.15E-07	6.15E-08	1.9
11	9.90E-08	5.60E-08	1.8

Supplementary Table 2. Mixed ion transport experiment data for the K^+/Li^+ system. Only data corresponding to membranes exhibiting a K^+ flux at least one order of magnitude lower than that of the bare polyelectrolyte-coated AAO support (values above the gray bar) are included in the analysis.

Measurement	K^+ Flux (mol cm^{-2} min^{-1})	Li^+ Flux (mol cm^{-2} min^{-1})	K^+/Li^+
1	6.00E-10	1.8E-10	3.3
2	1.10E-09	3.10E-10	3.5
3	1.85E-09	5.50E-10	3.4
4	3.44E-08	1.71E-08	2.0
5	3.90E-08	1.78E-08	2.2
6	6.56E-08	3.20E-08	2.1
7	1.27E-07	9.27E-08	1.4
8	9.88E-08	8.14E-08	1.2
9	1.15E-07	1.16E-07	1.0
10	1.73E-07	1.84E-07	0.9

Supplementary Table 3. Mixed ion transport experiment data for the K^+/Mg^{2+} system. Only data corresponding to membranes exhibiting a K^+ flux substantially lower than that of the bare polyelectrolyte-coated AAO support (values above the gray bar) are included in the analysis.

Measurement	K^+ Flux (mol cm^{-2} min^{-1})	Mg^{2+} Flux (mol cm^{-2} min^{-1})	K^+/Mg^{2+}
1	1.05E-08	<3.50E-11	>300
2	2.43E-08	2.60E-10	93.4
3	4.91E-08	4.50E-10	109.0
4	1.11E-07	9.71E-09	11.5
5	1.28E-07	1.38E-08	9.3

Supplementary Table 4. Water permeance and salt rejection of crown ether–based polyamide films formed directly on a porous PAN support under the same interfacial polymerization conditions used for freestanding films (DAB18C6 aqueous phase at 0.2 wt% in 20/80 DMF/water and TMC organic phase at 0.2 wt% in hexane), using a 5 min reaction time. Measurements were performed in a Millipore stirred cell pressurized to 3.5 bar. Rejection values are reported for single-salt tests with 0.1 M LiCl and 0.1 M KCl solutions, as well as for a mixed-salt feed containing 0.1 M KCl and 0.1 M $MgCl_2$.

Feed	Water Permeance (L m^{-2} h^{-1} bar^{-1})	KCl rejection (%)	LiCl rejection (%)	$MgCl_2$ rejection (%)
Single-salt (KCl 0.1M or LiCl 0.1M)	1.3 ± 0.2	10.1 ± 0.9	9.3 ± 1.2	--
Mixed-salt (KCl 0.1M + $MgCl_2$ 0.1M)	1.4 ± 0.3	8.6 ± 2.6	--	32.2 ± 0.6

Supplementary Table 5. Comparison of K^+/X^+ selectivity (where X^+ denotes other monovalent cations) and K^+ flux for the ultrathin crown ether–based polyamide membrane and reported membranes.

**Membranes**	**Feed solution (M)**	**K^+/X^+ selectivity**	**X^+**	**K^+ flux (mol cm^{-2} min^{-1})**	**Refs**
MXene	0.2	9	Na^+	1.7E-07	Supplementary Ref. 1
Prussian blue AAO	0.1	4	Li^+	1.2E-07	Supplementary Ref. 2
Polysulfone Gr	0.5	2.6	Na^+	1.0E-06	Supplementary Ref. 3
DB15C5@UiO-66	0.1	2, 3.5	Na^+, Li^+	1.2E-06	Supplementary Ref. 4
$TpBDMe_2$	0.1	4	Li^+	3.3E-07	Supplementary Ref.5
FGOM-60	0.1	2.3	Na^+	3.8E-09	Supplementary Ref. 6
PIM-BzMA-TB	1	1	Na^+	2.0E-06	Supplementary Ref. 7
DMBP-TB	1	3.3	Na^+	3.3E-07	Supplementary Ref. 7
Physically confined GO membrane	0.1	1.5	Na^+	1.1E-08	Supplementary Ref. 8
GO-PEI	1	1.3, 1.6	Na^+, Li^+	7.9E-07	Supplementary Ref. 9
r-GO	0.1	4, 10	Na^+, Li^+	3.0E-07	Supplementary Ref. 10
Layer-by-layer crown ether-based polyamide	0.1	1–2	Li^+, Cs^+	4.0E-07	Supplementary Ref. 11
Telechelic polymer with terminal crown ether	0.1	105	Na^+	2.0E-07	Supplementary Ref. 12
DB18C6-based polyamide via interfacial polymerization	0.1	3.8, 4.4	Li^+, Cs^+	2.0E-09	This Work

Supplementary References

1. Lu, Z., Wu, H., Wei, Y. & Wang, H. A Matthew MXene (Ti3C2T) Lamellar Membrane as a Potassium-Sieving Amplifier. *Engineering* **42**, 213–222 (2024).
2. Li, Z.-Q., Wu, M.-Y., Ding, X.-L., Wu, Z.-Q. & Xia, X.-H. Reversible Electrochemical Tuning of Ion Sieving in Coordination Polymers. *Anal. Chem.* **92**, 9172–9178 (2020).
3. Li, S., Lee, J.-H., Hu, Q., Oh, T.-S. & Yoo, J.-B. Scalable graphene composite membranes for enhanced ion selectivity. *Journal of Membrane Science* **564**, 159–165 (2018).
4. Xu, T. *et al.* Perfect confinement of crown ethers in MOF membrane for complete dehydration and fast transport of monovalent ions. *Sci. Adv.* **10**, eadn0944 (2024).
5. Sheng, F. *et al.* Efficient Ion Sieving in Covalent Organic Framework Membranes with Sub-2-Nanometer Channels. *Advanced Materials* **33**, 2104404 (2021).
6. Qian, Y. *et al.* Enhanced Ion Sieving of Graphene Oxide Membranes via Surface Amine Functionalization. *J. Am. Chem. Soc.* **143**, 5080–5090 (2021).
7. Tan, R. *et al.* Hydrophilic microporous membranes for selective ion separation and flow-battery energy storage. *Nat. Mater.* **19**, 195–202 (2020).

8. Song, J., Yu, H.-W., Ham, M.-H. & Kim, I. S. Tunable Ion Sieving of Graphene Membranes through the Control of Nitrogen-Bonding Configuration. *Nano Lett.* **18**, 5506–5513 (2018).
9. Huang, Q., Liu, S., Guo, Y., Liu, G. & Jin, W. Separation of mono-/di-valent ions via charged interlayer channels of graphene oxide membranes. *Journal of Membrane Science* **645**, 120212 (2022).
10. Xi, Y.-H. *et al.* Graphene-based membranes with uniform 2D nanochannels for precise sieving of mono-/multi-valent metal ions. *Journal of Membrane Science* **550**, 208–218 (2018).
11. Zhang, J., Villalobos, L. F., Lee, J., Zhong, M. & Elimelech, M. Ionophore-Based Molecular Layer-by-Layer Polyamide Membranes for Facilitated Single-Ion Transport. *ACS Appl. Mater. Interfaces* **17**, 30817–30824 (2025).
12. Li, C. *et al.* Biomimetic action-potential transmission via mechano-gated potassium channels assembled by quadruple hydrogen-bonded crown ethers. *Sci. Adv.* **12**, eaea6329 (2026).
